# Are there sex differences among colorectal cancer patients in treatment and survival? A Swiss cohort study

**DOI:** 10.1007/s00432-021-03557-y

**Published:** 2021-03-04

**Authors:** Manuela Limam, Katarina Luise Matthes, Giulia Pestoni, Eleftheria Michalopoulou, Leonhard Held, Silvia Dehler, Dimitri Korol, Sabine Rohrmann

**Affiliations:** 1 Epidemiology, Biostatistics and Prevention Institute, University of Zurich, Zurich, Switzerland; 2 Cancer Registry Zurich, Zug, Schaffhausen and Schwyz, University Hospital Zurich, Zurich, Switzerland

**Keywords:** Colorectal cancer, Sex differences, Comorbidity, Treatment, Survival

## Abstract

**Background:**

Colorectal cancer (CRC) is among the three most common incident cancers and causes of cancer death in Switzerland for both men and women. To promote aspects of gender medicine, we examined differences in treatment decision and survival by sex in CRC patients diagnosed 2000 and 2001 in the canton of Zurich, Switzerland.

**Methods:**

Characteristics assessed of 1076 CRC patients were sex, tumor subsite, age at diagnosis, tumor stage, primary treatment option and comorbidity rated by the Charlson Comorbidity Index (CCI). Missing data for stage and comorbidities were completed using multivariate imputation by chained equations. We estimated the probability of receiving surgery versus another primary treatment using multivariable binomial logistic regression models. Univariable and multivariable Cox proportional hazards regression models were used for survival analysis.

**Results:**

Females were older at diagnosis and had less comorbidities than men. There was no difference with respect to treatment decisions between men and women. The probability of receiving a primary treatment other than surgery was nearly twice as high in patients with the highest comorbidity index, CCI 2+, compared with patients without comorbidities. This effect was significantly stronger in women than in men (*p*-interaction = 0.010). Survival decreased with higher CCI, tumor stage and age in all CRC patients. Sex had no impact on survival.

**Conclusion:**

The probability of receiving any primary treatment and survival were independent of sex. However, female CRC patients with the highest CCI appeared more likely to receive other therapy than surgery compared to their male counterparts.

**Supplementary Information:**

The online version contains supplementary material available at 10.1007/s00432-021-03557-y.

## Background

About 4000 cases of colorectal cancer (CRC) are diagnosed in Switzerland every year, making it one of the three most common incident cancers, which is in line with the world cancer incidence report (Forman et al. 2013). Despite a decreasing mortality for both men and women, as recently reported for Switzerland (Bordoni et al. 2012), this cancer entity is still within the three most common causes of cancer death.

Investigations into differences of biological, behavioral and environmental factors between men and women and their impact on the manifestation, mechanism, treatment and outcome of disease is the task of gender medicine. Cardiology is one of the medical specialties pioneering scientific work done to establish and finally apply sex differences in the development and prevention of cardiovascular diseases, the awareness and presentation of their symptoms, and the effectiveness of therapy. In recent years, investigations in France have shown that the risk of women of developing cardiovascular diseases has been underestimated (Mounier-Vehier et al. 2010). In addition, an increasing proportion of women younger than 60 years of age with an acute coronary syndrome has been observed (Puymirat et al. 2012). Therefore, a gynecologist and a cardiologist working in the University hospital of Lille in France have introduced a care pathway “heart, arteries and women” for women with high cardiovascular risk (Mounier-Vehier et al. 2014). According to the recommendations of the American Heart Association (AHA) and the European Society of Cardiology, women should be carefully followed in their three phases of hormonal life: contraception, pregnancy and perimenopause.

In oncology, despite differences between sexes with respect to incidence rates, clinical presentation and prognosis have been described, notably for CRC, no comparable activities in patient care have been set up to the best of our knowledge. More men than women are diagnosed and die from CRC each year (Forman et al. 2013; Lorez et al. 2016). Among CRC patients, women are diagnosed at an older age (Brenner et al. 2007; Paulson et al. 2009) and on average, men are 4–6 years younger at time of death than women (Brenner et al. 2007). Women appear to have a higher risk of developing right-sided CRC than men (Hansen and Jess 2012; Kim et al. 2015; Lee et al. 2015). Several studies have observed an overall survival advantage for women with CRC (McArdle et al. 2003; Paulson et al. 2009; Storli et al. 2011; Wichmann et al. 2001). Comorbidities, diseases mostly diagnosed in older populations, are known to adversely influence treatment decisions and survival in cancer patients in general (Sarfati et al. 2016; Sogaard et al. 2013; Vulto et al. 2006) and in CRC patients (Janssen-Heijnen et al. 2005b; Lemmens et al. 2005a; Munro and Bentley 2004; Sarfati et al. 2009). A study in the Netherlands revealed that among elderly patients with stage III colon cancer diagnosed between 1995 and 2001 females and those with comorbidities were less likely to receive adjuvant treatment (Lemmens et al. 2005b). In a US study, women underwent less aggressive medical therapy in addition to surgery like neoadjuvant and/ or adjuvant treatment for advanced stage rectal and colon cancer than men, particularly in the octogenarian population (Paulson et al. 2009).

Cardiovascular diseases have been categorized for a long time as typically masculine and are often misdiagnosed in women (Hayes 2006). In contrast, pathognomonic symptoms for CRC are probably equally recognized in both men and women. Validated sex differences in cardiovascular diseases which affect men and women have been translated into improved medical prevention and disease care. CRC is also a common disease for which targeted investigations could lead to validation in sex differences to include them in guidelines and disease management. To support aspects of gender medicine, we investigated if differences in primary treatment option and survival by sex exist in Switzerland. To do so, we analyzed cancer registry data of 586 men and 490 women diagnosed with CRC in the years 2000 and 2001.

## Methods

### Study population

The Cancer Registry Zurich, founded in 1980, is an epidemiological tumor registry. Since 1980, all incident cases of cancer in the resident population of the canton of Zurich have been recorded. The canton of Zug joined the registry in 2011; Schaffhausen and Schwyz in 2020. This analysis only used data of the canton of Zurich. Systematically registered variables are cancer site, type of cancer, name, date of birth, sex, place of residence and last date of follow up. The registration of tumor stage, type of therapy and comorbidities was not mandatory for the incidence years in our study.

We extracted 1186 patients diagnosed with CRC as first primary cancer in the years 2000 and 2001 in the canton of Zurich. We excluded all patients registered as death certificate only cases (*N* = 69, 5.8%), cases identified at autopsy (*N* = 37, 3.1%) and cases with no specified colon site (*N* = 4, 0.3%). Finally, 1076 CRC cases were included in our study. The CRC cases were classified as C18-C20, based on the ICD-10 key system (International Statistical Classification of Diseases and Related Health Problems). Tumor stage was classified according to the 5th edition of the TNM classification system of the International Union Against Cancer. When no information was available on distant metastasis, we assumed that none existed. The M-category was defined as M zero (Sobin et al. 2009).

We defined two subsites, i.e., proximal or right-sided colon cancer (RCC) and distal or left-sided colorectal cancer (LCRC). RCC consists of tumors of the caecum (C18.0), colon ascendens (C18.2), flexura hepatica (C18.3) and colon transversum (C18.4). LCRC consists of tumors of the flexura lienalis (C18.5), colon descendens (C18.6), colon sigmoideum (C18.7), recto-sigmoid-junction (C19) and rectum (C20) (Bufill 1990; Hansen and Jess 2012; Lorez et al. 2016).

The following reasons led to the decision to categorize treatment into surgery and other primary treatment: the low number of cases and the fact that in 2014 optimal surgical resection is still the mainstay in curative CRC treatment, preferably in a multi-modal approach to maintain long-term-survival (Nakayama et al. 2013). Any surgical treatment was included in the surgery group while radiotherapy, radio-chemotherapy, chemotherapy and non-tumor-specific therapy were assigned to other primary treatment. Non-tumor-specific therapy was used when no surgery, radio- and/or chemotherapy were applied. Only the first treatment delivered was included in the analyses. Due to the lack of information on therapy intention, we were not able to define whether the first treatment applied was curative, more specifically neoadjuvant, or palliative. Three age groups were chosen for data analysis (< 65, 65–74 and ≥ 75). We obtained patients’ vital status from the Citizen Services Departments of the Canton of Zurich. Vital status was recorded until death or for a maximum period of 10 years after diagnosis.

As an additional impact factor, we used the Charlson Comorbidity Index (CCI). Charlson et al. (1987) have developed a classifying system for comorbidities in the form of a weighted index with the purpose to estimate the risk of mortality by taking number and severity of comorbid condition into account. For the original CCI, 19 conditions were defined and a weight as a point score from 1 to 6 was assigned to each of the conditions based on their rounded adjusted relative risk for 1-year mortality. The CCI is the sum of the weights for all concomitant conditions of a patient. A higher CCI score indicates a higher risk of mortality within 1 year. The weighted comorbidity index is the most widely used comorbidity scoring system in health research and in clinical practice, because it quantifies the individual’s disease burden and predicts hospital mortality in an easy way. Many studies have consistently shown that the CCI is a valid prognostic indicator for mortality (Goldstein et al. 2004; Lee et al. 2005; Myers et al. 2009). In our study, we only took into account the comorbidities present at the time of the CRC diagnosis. The CCI consisted of only 15 conditions, since CRC was the disease of interest and the cancer-related conditions were not taken into account. The points assigned to each of the recorded comorbidities are presented in Table 1. We built three CCI groups adapted to the size of our study population. The point scores for each patient were summed up and used to assign the patient to one of the groups: CCI 0 = no comorbidities, CCI 1 = sum of scores equal to 1, CCI 2+  = sum of scores equal to 2 or larger.

**Table 1:** Number of each comorbidity at diagnosis overall and stratified by sex

Comorbidities (corresponding weight [point score] for the CCI)	All	Male	Female
Myocardial infarction (1)	49	36	13
Congestive heart failure (1)	27	19	8
Peripheral vascular disease (1)	41	27	14
Cerebrovascular disease (1)	43	27	16
Dementia (1)	18	10	8
Chronic lung disease (1)	36	31	5
Connective tissue disease (1)	7	2	5
Peptic ulcer disease (1)	29	17	12
Mild liver disease (1)	13	8	5
Diabetes without target organ damage (1)	61	40	21
Hemiplegia (2)	7	3	4
Moderate to severe renal disease (2)	24	13	11
Diabetes with target organ damage (2)	19	16	3
Moderate to severe liver disease (3)	7	6	1
AIDS (6)	0	0	0
Total	381	255	126

*CCI* Charlson Comorbidity Index: the sum of the assigned weights results in the Index for a patient

We obtained information on tumor stage, type of therapy and comorbidity from patient records archived in the registry and from the hospital and the patient’s physician.

### Statistical methods

In our data set, 60 (5.6%) patients had missing information on the T-category, 79 (7.3%) on the N-category and 312 (29.0%) had missing information on comorbidities. We had no missing information on treatment. Obtaining information on comorbidities was limited by the time of diagnosis in 2000 and 2001, since medical records in Switzerland are often only stored until 10 years after the patient’s death, or the last visit at the practitioner or hospital. To overcome missingness, we used a standard multivariate imputation by chained equations (mice). This method took all variables into account, including the outcome variables: sex, date of diagnosis, age at diagnosis, survival time, vital status, tumor subsite and primary treatment, and the incomplete variables T-category, N-category and CCI (Moons et al. 2006). We created 30 imputed data sets using 10 iterations (Bodner 2008; White et al. 2011).

Studies showed that comorbidity leads to poorer survival in cancer patients in general (Janssen-Heijnen et al. 2005a; Sarfati et al. 2016; Sogaard et al. 2013) as well as in CRC patients (Boakye et al. 2018; Lemmens et al. 2005a). We found that patients without information on CCI had almost twice the risk of dying than patients with information on comorbidity (Supplementary material: Fig. 1S). Consequently, we have suspected that the probability of missingness depends on patients’ survival and on the unobservable value itself, which means that patients with a higher CCI are likely to have more missing information on comorbidity. Therefore, we assumed that missingness of comorbidity status is MNAR (missing not at random). We performed a sensitivity analysis to evaluate the influence of the unobserved values using ẟ-adjustment of the imputed data (Van Buuren 2018). Additionally, the results were compared with a complete-case analysis and post hoc sensitivity analysis. Comparison led to similar results and, consequently, we presented only the results obtained using multivariate imputation. After the imputation, we built the TNM tumor stage (I, II, III or IV) for each case from the TNM-categories.

With multivariable binomial logistic regression models we estimated the probability of receiving surgery versus another primary treatment depending on sex, subsite, CCI, tumor stage and age. Univariable and multivariable Cox proportional hazards regression models stratified by sex were performed to assess the effects of age, subsite, CCI, tumor stage and primary treatment option on survival. Since the biological variable sex is at the center of interest, we investigated interaction effects of sex in both the logistic and the Cox regression models. In the binomial logistic regression models, we evaluated the effect of sex on the association of subsite, CCI, tumor stage, age with primary treatment option. In the Cox regression models, we assessed if the impact of our exposure variables on survival depended on sex.

We used the R Version 3.6.0 for all statistical analyses. The R package “mice” (van Buuren and Groothuis-Oudshoorn 2011) was used to impute the missing data, the package “nnet” to perform the binomial logistic regression models (Venables and Ripley 2002), “survival” (Therneau 2015) to perform the Cox regression models. The plotting package “ggplot2” was used to visualize analyses (Wickham 2016).

## Results

**Fig. 1:**
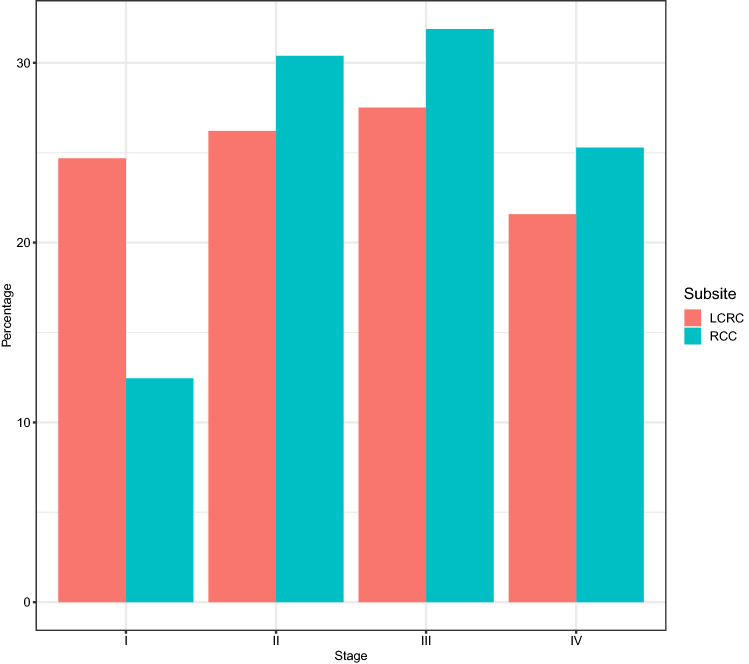
Colorectal cancer subsite distribution grouped by tumor stages (I–IV). *RCC* Right-sided colon cancer, *LCRC* Left-sided colorectal cancer

Table 2 illustrates the characteristics of 586 men and 490 women with CRC overall and by sex. LCRC was more common in males (57%) and RCC in females (52%). More men than women were diagnosed at an early tumor stage I or II (57%). Half as many RCC cases as LCRC cases were diagnosed at tumor stage I. At tumor stages II, III and IV more RCC cases were diagnosed (Fig. 1). Median age at diagnosis was higher in women (73 years) compared to their male counterparts (69 years). In the age group ≥ 75 years more female than male CRC patients were observed (55% versus 45%).

**Table 2:** Colorectal cancer patient characteristics^a^

	Total	Male				Female			
		All	CCI (Charlson Comorbidity Index)^c^			All	CCI (Charlson Comorbidity Index)^c^		
			0	1	2+		0	1	2+
Tumor site, *n* (%)									
C18–C20	1076 (100)	586 (54.46)	348 (59.39)	122 (20.82)	116 (19.80)	490 (45.54)	349 (71.22)	85 (17.35)	56 (11.43)
C18	672 (100)	367 (54.61)	205 (55.86)	83 (22.62)	79 (21.53)	305 (45.39)	211 (69.18)	56 (18.36)	38 (12.46)
C19	132 (100)	66 (50.00)	41 (62.12)	13 (19.70)	12 (18.18)	66 (50.00)	49 (74.24)	11 (16.67)	6 (9.09)
C20	272 (100)	153 (56.25)	102 (66.67)	26 (16.99)	25 (16.34)	119 (43.75)	89 (74.79)	18 (15.13)	12 (10.08)
RCC	331 (100)	158 (47.73)	83 (52.53)	38 (24.05)	37 (23.42)	173 (52.27)	105 (60.69)	40 (23.12)	28 (16.18)
LCRC	745 (100)	428 (57.45)	265 (61.92)	84 (19.63)	79 (18.46)	317 (42.55)	244 (76.97)	45 (14.20)	28 (8.83)
Age group, *n* (%)									
< 65 years	343 (100)	205 (59.77)	157 (76.59)	26 (12.68)	22 (10.73)	138 (40.23)	122 (88.41)	10 (7.25)	6 (4.35)
65–74 years	333 (100)	202 (60.66)	109 (53.96)	47 (23.27)	46 (22.77)	131 (39.34)	94 (71.76)	22 (16.79)	15 (11.45)
≥ 75 years	400 (100)	179 (44.75)	82 (45.81)	49 (27.37)	48 (26.82)	221 (55.25)	133 (60.18)	53 (23.98)	35 (15.84)
Median age, years (Q1/Q3)	70 (62/78)	69 (61/77)				73 (63/80)			
Stage, *n* (%)									
I	245 (100)	148 (60.41)	90 (60.81)	28 (18.92)	30 (20.27)	97 (39.59)	72 (74.23)	15 (15.46)	10 (10.31)
II	292 (100)	157 (53.77)	94 (59.87)	29 (18.47)	34 (21.66)	135 (46.23)	95 (70.37)	22 (16.30)	18 (13.33)
III	309 (100)	164 (53.07)	102 (62.20)	35 (21.34)	27 (16.46)	145 (46.93)	102 (70.34)	27 (18.62)	16 (11.03)
IV	230 (100)	117 (50.87)	62 (52.99)	30 (25.64)	25 (21.37)	113 (49.13)	80 (70.80)	21 (18.58)	12 (10.62)
Primary treatment, *n* (%)									
Surgery	921 (100)	498 (54.07)	298 (59.84)	106 (21.29)	94 (18.88)	423 (45.93)	304 (71.87)	73 (17.26)	46 (10.87)
Other^b^	155 (100)	88 (56.77)	50 (56.82)	16 (18.18)	22 (25.00)	67 (43.23)	45 (67.16)	12 (17.91)	10 (14.93)

*CCI* Charlson Comorbidity Index, *RCC* Right-sided colon cancer, *LCRC* Left-sided colorectal cancer

^a^Categorial variables are given as numbers (%); median age is a continuous variable given in years (Q1/Q3)

^b^Radiotherapy, radio-chemotherapy, chemotherapy or non-tumor-specific therapy

^c^CCI groups (0, 1, 2+) are indicated within sex

59% of the male patients and 71% of the female patients had no comorbidities. Overall, women had fewer comorbidities than men, even at an older age. For both men and women more than a third of the observed comorbidities were cardiovascular diseases including myocardial infarction, congestive heart failure, peripheral vascular disease and cerebrovascular disease. In descending order, men were also frequently affected by diabetes without target organ, chronic lung disease and peptic ulcer disease. In women, other frequent comorbidities were diabetes without target organ, peptic ulcer and moderate to severe renal disease (Table 1).

Table 3 shows the probability of receiving surgery versus another primary treatment depending on sex, subsite (LCRC versus RCC), CCI, tumor stage and age. Primary treatment option was dependent on subsite and CCI. For patients with a CCI 2+ the probability of receiving primary treatment other than surgery was nearly twice as high [CCI 2+ : odds ratio (OR) = 1.86 (95% confidence interval (CI) 1.08–3.23)] as for CRC patients with CCI 0. We observed a statistically significant interaction effect for sex on the association between CCI and primary treatment option (*p*-interaction = 0.010; Table 3), such that the effect of the association between having a CCI of 2+ and receiving other primary treatment than surgery was stronger in women [OR = 2.56 (95% CI 0.94–6.98)] than in men [OR = 1.54 (95% CI 0.78–3.04)]. All patients with RCC were significantly less likely to receive another primary treatment [OR 0.25 (95% CI 0.15–0.42)] than surgery. The treatment option did not depend on tumor stage and age. Female patients had the same chance as male patients of receiving any primary treatment option [OR 1.03 (95% CI 0.72–1.49)].

**Table 3:** Multivariable binomial logistic regression models for primary treatment option overall and stratified by sex

	All			Male			Female			Interaction
	Number		Other^a^ versus surgery	Number		Other^a^ versus surgery	Number		Other^a^ versus surgery	
	Surgery	Other^a^	OR (95% CI)	Surgery	Other^a^	OR (95% CI)	Surgery	Other^a^	OR (95% CI)	*p* Value
Sex										
Male	498	88	1.00	–	–	–	–	–	–	–
Female	423	67	1.03 (0.72–1.49)	–	–	–	–	–	–	–
Subsite										
LCRC	609	136	1.00	351	77	1.00	258	59	1.00	
RCC	312	19	0.25 (0.15–0.42)	147	11	0.33 (0.17–0.64)	165	8	0.17 (0.08–0.39)	0.074
CCI										
0	602	95	1.00	298	50	1.00	304	45	1.00	
1	179	28	1.01 (0.55–1.83)	106	16	0.83 (0.39–1.77)	73	12	1.31 (0.52–3.35)	
2+	140	32	1.86 (1.08–3.23)	94	22	1.54 (0.78–3.04)	46	10	2.56 (0.94–6.98)	0.010
Stage										
I	206	33	1.00	124	20	1.00	82	12	1.00	
II	254	39	1.17 (0.66– 2.09)	135	23	1.19 (0.58–2.47)	119	17	1.16 (0.46–2.93)	
III	271	44	1.26 (0.73–2.16)	144	23	1.15 (0.57–2.32)	127	20	1.41 (0.60–3.30)	
IV	190	39	1.63 (0.95–2.76)	95	22	1.60 (0.80–3.18)	95	18	1.68 (0.72–3.94)	0.349
Age group										
< 65 years	290	53	1.00	174	31	1.00	116	22	1.00	
65–74 years	286	47	0.92 (0.59–1.44)	168	34	1.78 (0.67–2.06)	118	13	0.59 (0.27–1.26)	
≥ 75 years	345	55	0.94 (0.60–1.46)	156	23	0.89 (0.48–1.65)	189	32	0.93 (0.48–1.78)	0.451

*CI* Confidence interval*, LCRC* Left-sided colorectal cancer, *RCC* Right- sided colon cancer, *CCI* Charlson Comorbidity Index

^a^Radiotherapy, radio-chemotherapy, chemotherapy or non-tumor-specific therapy

**Fig. 2:**
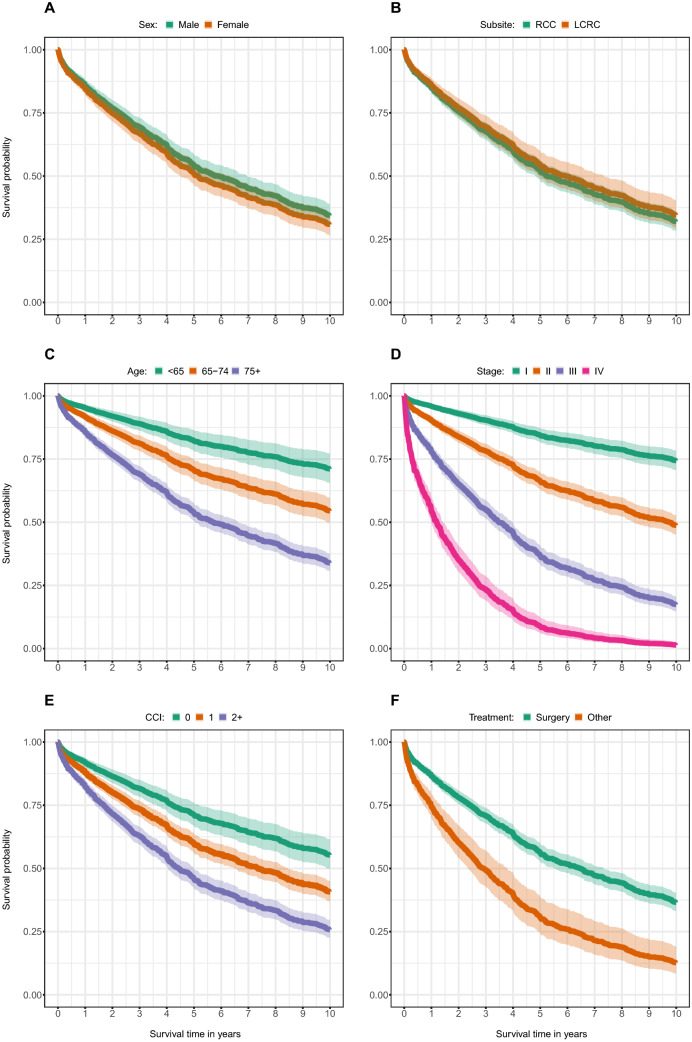
Adjusted Kaplan–Meier survival curves with confidence bands for CRC patients stratified by **a** sex, **b** subsite, **c** age, **d** tumor stage, **e** CCI (Charlson Comorbidity Index), **f** primary treatment option. All results were mutually adjusted for all variables

Next, we examined whether differences in survival existed between male and female CRC patients. Survival decreased with higher CCI, tumor stage and age in both men and women (Table 4; Fig. 2). CRC patients of either sex had basically twice the risk to die after another primary treatment than after surgery [Hazard ratio (HR) = 2.04 (95% CI 1.65–2.53)]. Sex had no impact on survival (Fig. 2).

**Table 4:** Univariable and multivariable Cox regression models for survival analysis overall and stratified by sex

	All		Male		Female		Interaction	
	Univariable	Multivariable	Univariable	Multivariable	Univariable	Multivariable	Univariable	Multivariable
	HR (95% CI)	HR (95% CI)	HR (95% CI)	HR (95% CI)	HR (95% CI)	HR (95% CI)	*p* Value	*p* Value
Sex								
Male	1.00	1.00	–	–	–	–	–	–
Female	1.14 (0.98–1.33)	1.04 (0.89–1.22)	–	–	–	–	–	–
Subsite								
LCRC	1.00	1.00	1.00	1.00	1.00	1.00		
RCC	1.25 (1.07–1.47)	1.02 (0.85–1.21)	1.20 (0.96–1.52)	1.02 (0.80–1.31)	1.27 (1.02–1.59)	1.03 (0.81–1.32)	0.750	0.928
CCI								
0	1.00	1.00	1.00	1.00	1.00	1.00		
1	1.96 (1.56–2.44)	1.57 (1.24–1.99)	2.14 (1.56–2.93)	1.66 (1.21–2.29)	1.88 (1.36–2.59)	1.51 (1.07–2.13)		
2+	2.07 (1.66–2.59)	2.02(1.58 –2.58)	2.42 (1.80 –3.24)	2.26 (1.65 –3.10)	2.11 (1.43–3.12)	1.73 (1.12–2.69)	0.761	0.309
Stage								
I	1.00	1.00	1.00	1.00	1.00	1.00		
II	1.85 (1.40–2.45)	1.76 (1.32–2.35)	2.01 (1.39–2.93)	1.99 (1.36–2.90)	1.64 (1.07–2.52)	1.51 (0.97–2.32)		
III	2.92 (2.23–3.82)	3.18 (2.43–4.17)	2.73 (1.91–3.91)	3.08 (2.13–4.43)	3.09 (2.08–4.62)	3.27 (2.18–4.89)		
IV	11.70 (8.88–15.30)	13.90 (10.45–18.56)	12.70 (8.80–18.30)	16.30 (11.0 -24.10)	10.40 (6.91–15.70)	11.80 (7.73–18.03)	0.949	0.623
Primary Treatment								
Surgery	1.00	1.00	1.00	1.00	1.00	1.00		
Other^a^	1.71 (1.40–2.09)	2.04 (1.65–2.53)	1.77 (1.36–2.31)	2.14 (1.61–2.85)	1.64 (1.22–2.23)	1.87 (1.35–2.58)	0.736	0.602
Age group								
< 65 years	1.00	1.00	1.00	1.00	1.00	1.00		
65–74 years	1.36 (1.09–1.68)	1.51 (1.21–1.88)	1.40 (1.06–1.84)	1.49 (1.12–2.00)	1.29 (0.92–1.81)	1.51 (1.07–2.13)		
≥ 75 years	2.85 (2.35–3.46)	3.10 (2.53–3.82)	2.68 (2.06–3.49)	2.78 (2.09–3.71)	2.99 (2.25–4.00)	3.48 (2.57–4.71)	0.433	0.216

*HR* Hazard ratio, *CI* Confidence interval, *LCRC* Left-sided colorectal cancer, *RCC* Right-sided colon cancer, *CCI* Charlson Comorbidity Index

^a^Radiotherapy, radio-chemotherapy, chemotherapy or non-tumor-specific therapy

## Discussion

We used the data of the largest cantonal cancer registry in Switzerland to evaluate differences in primary treatment option and survival between women and men diagnosed with CRC in 2000 and 2001. The results of our study indicate that in the Swiss Canton of Zurich female CRC patients had the same probability as male CRC patients of receiving surgery or another primary treatment. However, a statistically significant interaction effect indicated that sex influences the association between CCI and primary treatment option, such that the effect of the association between having a CCI 2+ and receiving other primary treatment than surgery was stronger in women than in men. Furthermore, in this patient cohort having made adjustments for age, subsite, CCI, tumor stage and primary treatment option, men and women with CRC had equal overall survival.

According to the World Health Organization (WHO) global report on prevention of chronic diseases published in 2006, chronic diseases affect men and women almost equally (Tunstall-Pedoe 2006). It is known that comorbidity is common in CRC patients (Sarfati et al. 2011). Our data are comparable with the worldwide cancer incidence and mortality report using data of the GLOBOCAN 2012 project, stating that the incidence of CRC in populations over 65 years of age is higher for women than for men (Ferlay et al. 2015). Female CRC patients are diagnosed at an older age compared with men as already shown in previous studies (Brenner et al. 2007; Paulson et al. 2009). At the same time, using the CCI, women in our patient cohort had a better underlying general state of health than men. Similarly, Katzenstein et al. (2018) observed that male rectal cancer patients developed a higher overall morbidity than women. Additionally, they reported a higher presence of risk factors such as alcohol and nicotine abuse, as well as higher body mass index (BMI) in male patients (Katzenstein et al. 2018). Cigarette smoking increases the risk of chronic diseases such as cardiovascular and chronic lung diseases but also of CRC (Johnson et al. 2013). Unfortunately, we did not have risk factor information available for our analysis and could not determine which factors were associated with the presence of comorbidity in men and in women. Future studies are needed to validate whether female CRC patients generally have a better health status than male CRC patients.

Our study revealed that the primary treatment option was dependent on CCI and subsite. In 2016, Sarfati et al., reviewed current knowledge of comorbidity in cancer patients and its impact on cancer diagnosis, treatment, and patient outcomes. They reported that cancer patients with comorbidity were generally less likely to receive curative treatment for their cancer than those without comorbidity (Sarfati et al. 2016). This is in line with other findings, in particular for CRC patients (Cronin et al. 2006; Gross et al. 2007; Khrizman et al. 2013; Lemmens et al. 2005a). In our study, although both treatment options, i.e., surgery and another primary treatment, contained both curative and palliative intentions, increasing CCI reduced the probability of surgical treatment. We hypothesize that increasing CCI reduced the probability of receiving curative surgical treatment. To address the challenges of comorbidity in cancer, Sarfati et al. (2016) proposed to improve the assessment of comorbidities among cancer patients and also to improve evidence from which to make cancer treatment decisions. This is particularly desirable, because cancer patients with comorbidity are often excluded from clinical trials leading to inconsistency in cancer treatment decisions. For cancer treatment decisions, it is common that simultaneously occurring comorbidities are considered as a “single-disease”, because the complex interrelations of cancer treatment and number, type and severity of comorbidities are mostly unknown. Our results indicate that biological sex influences the impact of CCI on primary treatment option. Hence, it would be interesting to examine the quality of comorbidity assessment and whether this differs between men and women. Moreover, knowledge about how different types of comorbidity interact with CRC and its treatment in men and in women could optimize treatment decisions.

Due to the number of patients, we stratified primary treatment options into two groups and observed that the probability of receiving a primary treatment other than surgery was higher for LCRC (including rectal cancers) than for RCC. In the last two decades of the twentieth century treatment strategy in rectal cancer has changed regarding surgical techniques and adjuvant therapy. Concerning adjuvant therapy several studies have found a lower recurrence rate after adding preoperative radiotherapy to surgery in patients with resectable rectal cancer (Gérard et al. 1988; Goldberg et al. 1994; Horn et al. 1990; Jones et al. 1989). Furthermore, a Swedish rectal cancer trial revealed in 1997 a significant increase in overall survival in patients who received preoperative radiotherapy followed by surgery compared to patients who received surgery only (Cedermark et al. 1997). Probably this supported the introduction of the recommendation to apply neoadjuvant radiotherapy in rectal cancer patients at least in some European countries such as the Netherlands and France (Martijn et al. 2003; NN 1995). To the best of our knowledge no Swiss guidelines in rectal cancer management existed at the incidence years 2000 and 2001. As we only considered the first therapy administered without information on treatment intention, we cannot say for certain whether radiotherapy as the first therapy applied for rectal cancer was neoadjuvant radiotherapy.

In our study, men and women had the same probability of receiving any primary treatment option. Only a few studies have investigated sex differences on treatment option in CRC patients. Paulson et al. reported that women with advanced tumor stage were treated with less aggressive medical therapy, especially at an older age (Paulson et al. 2009). Similarly, Lemmens et al. (2005a, b) observed that the odds of receiving adjuvant therapy for CRC patients with tumor stage III depended on age and comorbidity but also on sex, and that women were less likely to be treated with adjuvant chemotherapy (Lemmens et al. 2005b). However, in our findings women in the highest CCI group appeared more likely to receive other therapy than surgery in comparison to men. The reasons for this potential disadvantage in female patients with concomitant diseases to receive optimal CRC treatment are unclear. Further investigations should determine the factors influencing cancer treatment choice in both men and women. Also, this illustrates the importance of standardized comorbidity assessment in the cancer treatment decision process to better ensure optimal therapy.

In this patient cohort, having made adjustments for age, subsite, CCI, tumor stage and primary treatment option, we observed that men and women with CRC had equal overall survival. In the literature of the last two decades, findings concerning sex differences on survival of CRC patients are inconsistent. Katzenstein et al. (2018) found no sex differences in oncological long-term results for rectal cancer patients after surgery including total morbidity in the multivariable analysis. Lydrup et al. (2015) observed equal survival for men and women with rectal cancer after correction for the underlying mortality in the population knowing that women generally live longer than men. This is in contrast to most previous publications, which observed a survival advantage for women after surgical treatment (McArdle et al. 2003; Paulson et al. 2009; Storli et al. 2011; Wichmann et al. 2001). It is important to note that none of these investigations had taken comorbidities into account.

In line with our results, comorbidity has consistently been found to have an adverse impact on CRC survival (Gross et al. 2006; Munro and Bentley 2004; Rieker et al. 2002; Sarfati et al. 2009). A plausible reason why comorbidity in CRC patients influences survival might be the direct impact of the underlying conditions on mortality. Nevertheless, there might also be indirect reasons for this effect. We found a significant impact of comorbidity on survival using multivariable cox regression models. As discussed above, it has been shown that patients with comorbidity receive less curative cancer treatment than patients without comorbidity. This is also true for our study, where the chance of receiving surgical treatment decreased with increasing comorbidity. In addition, it is impossible to exclude an adverse impact of cancer and its treatment on comorbidity development and progression. For instance, a locally spreading tumor, paraneoplastic syndrome, chemotherapy or hormonal therapy could affect the risk of cardiovascular, metabolic, and other diseases and exacerbate preexisting comorbidities. The importance of a better understanding of the interrelations between cancer, its treatment and the comorbidities is obvious.

We observed that RCC patients were more likely to be female and had more advanced tumor stages. These observations are consistent with other studies (Benedix et al. 2010; Hansen and Jess 2012; Leijssen et al. 2018). We observed in the univariable analysis that all patients with RCC and women with RCC had worse survival than their respective counterparts with LCRC. However, after multivariable adjusting this effect was attenuated and no longer statistically significant. Similarly, Leijssen et al. observed no association between tumor location and survival after adjustment for several factors including pathological factors such as differentiation and microsatellite instability (Leijssen et al. 2018). This is in contrast with other studies including a meta-analysis that reported worse survival in patients with right-sided colon cancer than with left-sided colon cancer (Benedix et al. 2010; Yahagi et al. 2016).

Some limitations of our study have to be taken into account. Due to a reduced number of patients in several treatment groups, it was not possible to create more than two treatment categories in our analysis. We were also not able to include additional risk factors such as smoking, dietary habits or seeking medical advice. We had missing information on T-category, N-category and comorbidities. However, we used imputation methods and a comparison between a complete-case analysis and the imputed analysis revealed similar results. Finally, we would like to mention that immortal time bias cannot be excluded completely. Since we do not know the date when the treatment started, we do not know if the time period between diagnosis and start of treatment differed for the two treatment groups. The major strengths of our study were the data quality and the inclusion of the CCI. We used data from the largest cancer registry in Switzerland, where all incidence cases are registered with information on place of diagnosis, allowing us to add the parameters CCI, tumor stage and primary treatment option for the incidence years 2000 and 2001.

## Conclusion

In this patient cohort, after adjustment for sex, tumor subsite, CCI, tumor stage and age, women had the same chance as men to receive any primary treatment option for CRC cancer and overall survival did not differ by sex. However, comorbidity impacts survival and a further analysis revealed that the impact of comorbidity on primary treatment option depended on sex. Women in the highest CCI group appeared more likely to receive other therapy than surgery in comparison to men. Comorbidity is an important interacting and prognostic factor for CRC, but the complex interrelations between comorbidity, cancer and its treatment are still largely unknown. Here, targeted investigations by respecting sex should contribute to fill the gaps in modern individualized medicine resulting in improvement of prevention measures, treatment planning and outcome for male and female CRC patients.

## Supplementary Information

Below is the link to the electronic supplementary material.

Supplementary file 1: contains Fig. 1S Cumulative Baseline Hazard analysis depending on availability of information on comorbidity at diagnosis and its association with CRC-related mortality after 10 years. Patients without information on comorbidity had almost twice the risk of dying ten years after diagnosis than patients with information on comorbidity (PDF 13 KB)

## Data Availability

The datasets generated and/or analyzed during the current study are not publicly available due to individual privacy reasons, but are available from the corresponding author on reasonable request.

## Abbreviations

CRC: Colorectal cancer
CCI: Charlson Comorbidity Index
AHA: American Heart Association
ICD: International Classification of Diseases
TNM (Classification of malignant tumors): Tumor, lymph nodes, metastasis
RCC: Right-sided colon cancer
LCRC: Left-sided colorectal cancer
Mice: Multivariate imputation by chained equations
MNAR: Missing not at random
OR: Odds ratio
CI: Confidence interval
HR: Hazard ratio
WHO: World Health Organization
BMI: Body mass index
