# Supplementary figures and images for: Are there sex differences among colorectal cancer patients in treatment and survival? A Swiss cohort study
Source: J Cancer Res Clin Oncol. 2021 Mar 4;147(5):1407–19. doi: 10.1007/s00432-021-03557-y (PMC8021518; doi:10.1007/s00432-021-03557-y)

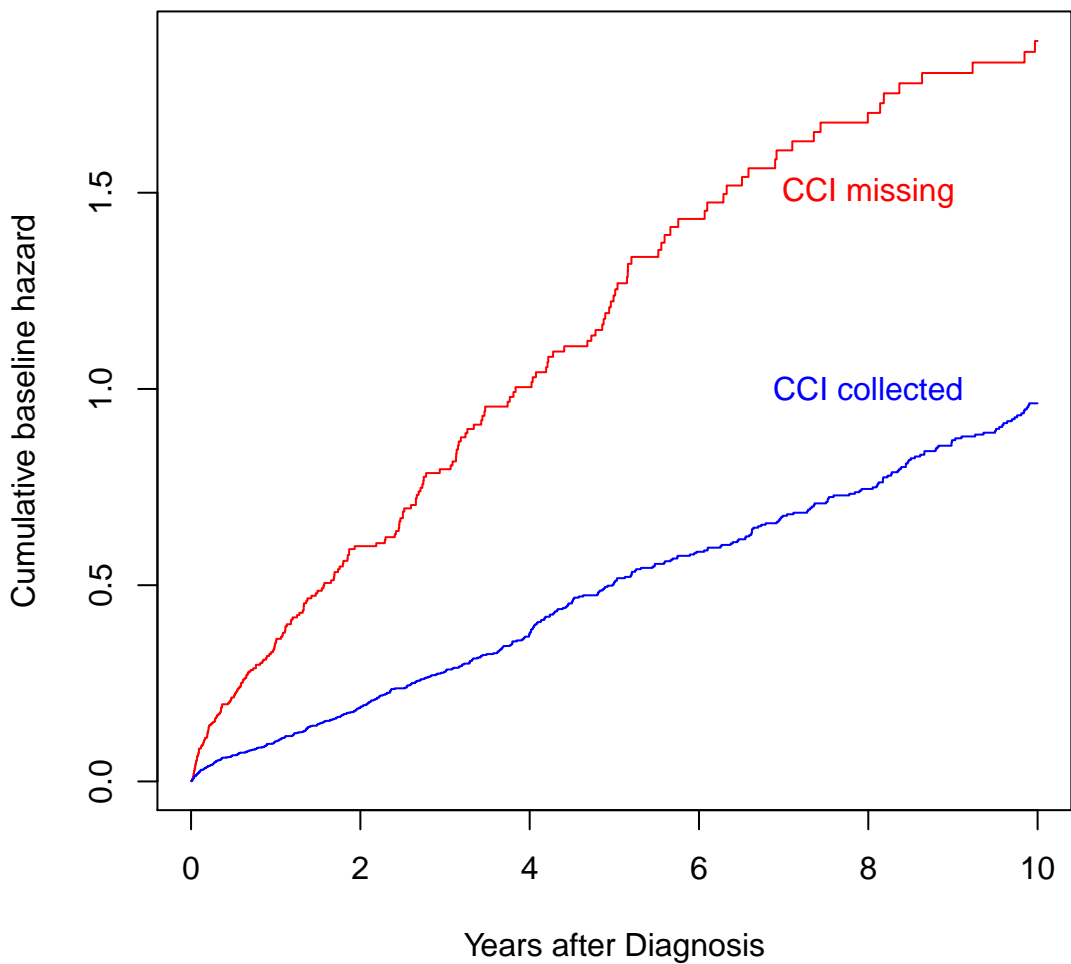

Supplement: Supplementary file 1 — contains Fig. 1S Cumulative Baseline Hazard analysis depending on availability of information on comorbidity at diagnosis and its association with CRC-related mortality after 10 years. Patients without information on comorbidity had almost twice the risk of dying ten years after diagnosis than patients with information on comorbidity (PDF 13 KB) [file 432_2021_3557_MOESM1_ESM.pdf]
